# Coronary Artery Restenosis in Women by History of Preeclampsia

**DOI:** 10.1161/JAHA.122.026287

**Published:** 2022-09-08

**Authors:** Annie Lin, Moa Pehrson, Giovanna Sarno, Abigail Fraser, Janet W. Rich‐Edwards, Isabel Gonҫalves, Mats Pihlsgård, Simon Timpka

**Affiliations:** ^1^ Perinatal and Cardiovascular Epidemiology Lund University Diabetes Centre, Clinical Sciences Malmö, Lund University Malmö Sweden; ^2^ Department of Medical Sciences Cardiology and Uppsala Clinical Research Center, Uppsala University Uppsala Sweden; ^3^ Population Health Science, Bristol Medical School University of Bristol Bristol United Kingdom; ^4^ Division of Women’s Health Department of Medicine, Brigham and Women’s Hospital and Harvard Medical School Boston MA; ^5^ Department of Cardiology and Cardiovascular Research Translational Studies Clinical Sciences Malmö, Lund University Malmö Sweden; ^6^ Department of Obstetrics and Gynecology Skåne University Hospital Malmö Sweden

**Keywords:** coronary artery disease, coronary artery stenting, hypertensive disorders of pregnancy, pregnancy, SCAAR, SWEDEHEART, Epidemiology, Percutaneous Coronary Intervention, Revascularization, Preeclampsia, Coronary Artery Disease

## Abstract

**Background:**

A history of preeclampsia is associated with increased risk of coronary artery disease and experimental evidence suggests that a history of preeclampsia also increases the risk of restenosis. However, the extent to which a history of preeclampsia is associated with risk of restenosis after percutaneous coronary intervention in women is unknown.

**Methods and Results:**

We included 6065 parous women aged ≤65 years with first percutaneous coronary intervention on 9452 segments 2006 to 2017, linking nationwide data on percutaneous coronary intervention and delivery history in Sweden. Main outcomes were clinical restenosis and target lesion revascularization within 2 years. We accounted for segment‐, procedure‐, and patient‐related potential predictors of restenosis in proportional hazards regression models. Restenosis occurred in 345 segments (3.7%) and target lesion revascularization was performed on 383 patients (6.3%). A history of preeclampsia was neither significantly associated with risk of restenosis (predictor‐accounted hazard ratio [HR], 0.71 [95% CI, 0.41–1.23]) nor target lesion revascularization (0.74 [95% CI, 0.51–1.07]) compared with a normotensive pregnancy history. When term and preterm preeclampsia were investigated separately, segments in women with a history of term preeclampsia had a lower risk of restenosis (predictor‐accounted HR, 0.45 [95% CI, 0.21–0.94]). A history of preeclampsia was not significantly associated with death by any cause within 2 years of the index procedure (predictor‐accounted HR 1.06, [95% CI, 0.62–1.80]).

**Conclusions:**

A history of preeclampsia was not associated with increased risk of restenosis but instead some evidence pointed to a decreased risk. To facilitate future studies and allow for replication, concomitant collection of data on pregnancy complication history and percutaneous coronary intervention outcomes in women is warranted.

**Nonstandard Abbreviations and Acronyms**
DES: drug‐eluting stent
MBR: Swedish Medical Birth Registry
SCAAR: Swedish registry for coronary angiography and angioplasty
Sflt‐1: soluble fms‐like tyrosine kinase‐1

**Clinical Perspective**

**What Is New?**

- A history of preeclampsia was not associated with increased risk of restenosis following percutaneous coronary intervention in women aged ≤65 years.
- Some evidence pointed to a decreased risk of restenosis in women with a history of preeclampsia at term.

**What Are the Clinical Implications?**

- There is insufficient evidence to clinically consider history of preeclampsia in women undergoing percutaneous coronary intervention.
- To facilitate future studies and allow for replication, concomitant collection of data on pregnancy complication history and percutaneous coronary intervention outcomes in women is warranted.

Despite significant progress during the past 2 decades with the introduction of drug‐eluting stents (DES) and devices,^1^, ^2^ restenosis remains as an important complication after percutaneous coronary intervention (PCI).^2^, ^3^ Compared with men, women have higher risk of needing target lesion revascularization (TLR)^4^ and of all‐cause mortality^5^ following PCI. If relevant female‐specific prognostic factors are identified, this sex‐disparity in outcomes might be reduced through improved care of women. One female‐specific risk factor for developing coronary artery disease is a history of preeclampsia, a hypertensive disorder of pregnancy^6^, ^7^ hallmarked by placental dysfunction. However, experimental and clinical evidence suggest that a history of preeclampsia, or placental dysfunction, is also associated with restenosis^8^ and mortality after revascularization.^9^

In an experimental model, preeclampsia in mice — induced through overexpressing a soluble vascular endothelial growth factor receptor — resulted in amplified vascular remodeling following vascular injury after the postpartum period, with increased vascular smooth cell proliferation and vascular fibrotic response.^8^ Imbalance between this antiangiogenic receptor, Sflt‐1 (soluble fms‐like tyrosine kinase‐1), and its ligands is considered to be a central pathway through which the maternal syndrome of preeclampsia occurs.^10^ Moreover, small clinical studies have implicated the same vascular endothelial growth factor‐related single nucleotide polymorphism in both severe preeclampsia^11^ and coronary artery restenosis.^12^ A study from Canada has also reported that women with a history of placental events — a term that includes preeclampsia — have higher mortality following revascularization.^9^

We therefore hypothesized that women with a history of preeclampsia have higher risk of restenosis and need of TLR following PCI. To test this hypothesis, we conducted a large nationwide study by merging data of the patient's PCI procedures with their preeclampsia history, using data ascertained via comprehensive clinical registries in Sweden.

## METHODS

**Figure 1:**
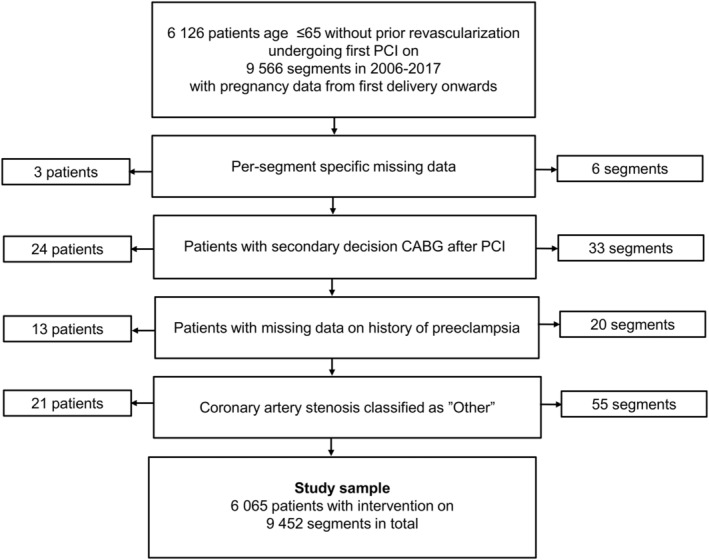
Flowchart of study sample. CABG indicates coronary artery bypass graft; and PCI, percutaneous coronary intervention.

We conducted a prospective cohort study linking data collected from 2 nationwide and comprehensive clinical registries in Sweden. The SCAAR (Swedish Registry for Coronary Angiography and Angioplasty) contains data from all Swedish centers performing PCI, whereas the MBR (Swedish Medical Birth Registry) contains data on the large majority of all deliveries in Sweden since 1973. As shown in Figure 1, our sample consisted of parous women aged ≤65 years who had their first delivery registered in MBR and a first PCI between May 2006 to 2017. We excluded patients with first PCI before 2006, planned or prior coronary artery bypass grafting (CABG), or missing data on any segment‐related variable. We further excluded patients (n=21) with a stenosis at the index procedure classified as “Other”. Data on migration following the index procedure were ascertained from Statistics Sweden. The study was approved by the Ethical Review Board in Lund, Sweden, and all patients gave informed consent to be included in the SCAAR registry. Because of the sensitive nature of the data collected for this study, requests to access the data from qualified researchers trained in human subject confidentiality protocols may be sent to SCAAR, the Swedish Board of Health and Welfare (MBR), and Statistics Sweden.

### Data on History of Preeclampsia

Data on exposure in any pregnancy to preeclampsia or nonpreeclamptic hypertension in pregnancy were ascertained from the MBR. The register started in 1973 and includes data on almost all pregnancies leading to delivery in Sweden.^13^ For this study preeclampsia and nonpreeclamptic hypertension in pregnancy were defined in accordance with *International Classification of Diseases, Eighth, Ninth, and Tenth Revisions (ICD‐8, ICD‐9, and ICD‐10)*. Diagnoses were set according to clinical practice at time of diagnosis and pre‐existing hypertension was also registered early in pregnancy. For further information on specific diagnoses, see Data S1. In this study, hypertensive exposure in pregnancy was divided into 3 categories: normotensive, nonpreeclamptic hypertension in pregnancy (gestational hypertension and essential hypertension), and preeclampsia. Preeclampsia was further categorized into 2 different subgroups: preterm preeclampsia (with delivery gestational week ≤36+6) and term preeclampsia (with delivery gestational week 37+0 or later). Essential hypertension in early pregnancy with diagnosed preeclampsia was defined as preeclampsia. Because of the exposures being rare, gestational hypertension and essential hypertension were combined in the category nonpreeclamptic hypertension in pregnancy to separate from normotensive pregnancies. Parity was defined as the total number of deliveries registered in MBR.

### Data on PCI

Data on patient characteristics and peri‐procedural variables were ascertained from SCAAR, which contains data on all coronary angiography and PCI procedures performed in Sweden. The following variables were included in this study as procedural predictors of restenosis: indication for procedure, stented vessel, type of device(s) used, length of stent (if used), and stent diameter following procedure. We further included several patient‐related predictors for restenosis at the time of stenting: diabetes, smoking, hypertension, dyslipidemia, and previous myocardial infarction. To account for the general improvement of care during the study period, we also included a categorical variable indicating year of procedure (2006–2009, 2010–2013, or 2014–2017).

### End Point Definitions

In the restenosis analysis the primary end point was clinical restenosis, which is routinely collected in SCAAR. We defined restenosis according to the definition in the SCAAR registry: a stenosis assessed by angiographic visual estimation (>50%) or by fractional flow reserve ≤0.80 in a previously stented segment identified by coronary angiography for any clinical indication performed anywhere in the country.^14^, ^15^

The TLR was defined as either any repeat PCI on a segment targeted at the index procedure or CABG following the index PCI, whichever occurred first. New PCI was ascertained via the SCAAR registry and CABG procedures (operational codes FNC and FND) via the Swedish in‐patient registry, a comprehensive national registry of inpatient care. To ensure that CABG surgeries already planned at the time of index procedure where not included as end points, we excluded patients with a secondary decision of CABG at index PCI as described above.

To analyze the end point death by any cause, we defined the event of death as registered in the comprehensive Swedish cause of death register.

### Statistical Analysis

We performed 2 complementary sets of analyses, investigating both time to restenosis per‐segment and time to TLR per patient by history of preeclampsia. The former analyses allow for sophisticated consideration of peri‐procedural characteristics as the targeted segment is the unit of analysis, whereas the latter is the equivalent per‐patient analysis. We used Kaplan–Meier curves to plot time‐to‐event by history of preeclampsia and proportional hazard regression to estimate hazard ratios (HR) and account for plausible predictors of restenosis. The start of the study time in 2006 corresponds to when all covariables were routinely collected in SCAAR. All *P* values are 2‐sided, *P*<0.05 was considered notable. All analyses were performed using SAS version 9.4.

For the per‐segment analyses, we used Cox regression with a jackknife estimator of variance to account for the statistical dependence between coronary artery segments in the same patient. To account for all type of device combinations (eg, DES preceded by dilation with balloon or not) and include them in 1 coherent analysis, we combined appropriate interaction terms of the 3 binary variables drug‐eluting, stent, and balloon. This allowed us to account for other stent‐related features (eg, stent length) by including an interaction term with stent. All per‐segment models were censored at 2 years of follow‐up, end of follow‐up, death, or migration, whichever occurred first.

For the per‐patient TLR analysis, we used standard proportional hazard regression and accounted for all predictors on a patient level. To use information on the segment level in the per‐patient analyses, we allowed for several possible alternatives within a single patient for the variable treated vessel (ie, each treated vessel category was included as a not mutually exclusive binary variable). We also further accounted for the variable number of treated vessels in the TLR analysis. All models were censored at 2 years, end of follow‐up, death, or migration, whichever occurred first.

For the mortality analysis, we used proportional hazards regression accounted for age and censored at 2 years, end of follow‐up, or migration, whichever came first. The small number of events prevented full consideration of the predictors included in the TLR analyses.

We checked the proportional hazards assumption using graphical methods and tests of Schoenfeld residuals, which indicated no violations of model assumptions.

### Multiple Imputation

We used multiple imputation to address missing covariable data on the patient level. We used multiple imputation by chained equations with 20 imputed data sets and estimates were combined by applying PROC MIANALYZE in SAS 9.4. As described above, patients with missing data on any segment‐level variable were excluded; 5.7% of the segments had missing data on at least 1 covariable. Data on preeclampsia exposure and end points were not imputed. For more information on missing data per variable see Table 1.

**Table 1:** Descriptive Characteristics Per Segment (n=9452) at the Index PCI Procedure by Preeclampsia History

				Subgroups of Preeclampsia		
Variables, n (%) unless stated	Normotensive (n=8111)	Nonpreeclampsia hypertension in pregnancy (n=432)	Preeclampsia (n=909)	Term preeclampsia (n=636)	Preterm preeclampsia (n=273)	Missing (%)
Patient characteristics						
Age (SD)	55.4 (6.4)	54.5 (6.7)	52.8 (7.3)	54.1 (6.7)	50.0 (7.8)	0.0
Parity						
1	1798 (22.2)	84 (19.4)	157 (17.3)	110 (17.3)	47 (17.2)	0.0
2	3684 (45.4)	221 (51.2)	410 (45.1)	282 (44.3)	128 (46.9)	0.0
3 or more	2629 (32.4)	127 (29.4)	342 (37.6)	244	98 (35.9)	0.0
Previous MI	327 (4.1)	30 (7.1)	38 (4.2)	15 (2.4)	23 (8.6)	1.5
Diabetes	1222 (15.2)	107 (25.1)	237 (26.2)	139 (22.0)	98 (35.9)	0.6
Hypertension	3414 (42.8)	285 (67.2)	559 (62.0)	390 (62.0)	169 (61.9)	1.7
Dyslipidemia	2511 (31.6)	161 (38.4)	322 (35.9)	212 (33.9)	110 (40.6)	1.9
Smoking						3.7
Never	2200 (28.1)	177 (43.5)	361 (41.1)	252 (40.9)	109 (41.4)	
Ex‐smoker	1873 (24.0)	106 (26.0)	248 (28.2)	181 (29.4)	67 (25.5)	
Smoker	3744 (47.9)	124 (30.5)	270 (30.7)	183 (29.7)	87 (33.1)	
Procedural characteristics						
Year of index PCI						0.0
2006–2009	1988 (24.5)	143 (33.1)	227 (25.0)	150 (23.6)	77 (28.2)	
2010–2013	2647 (32.6)	143 (33.1)	294 (32.3)	213 (33.5)	81 (29.7)	
2014–2017	3476 (42.9)	146 (33.8)	388 (42.7)	273 (42.9)	115 (42.1)	
Indication for PCI						0.0
STEMI	2891 (35.6)	157 (36.3)	291 (32.0)	211 (33.2)	80 (29.3)	
NSTEMI	1190 (14.7)	44 (10.2)	120 (13.2)	91 (14.3)	29 (10.6)	
Unstable CAD	2526 (31.1)	125 (28.9)	310 (34.1)	217 (34.1)	93 (34.1)	
Stable CAD	1255 (15.5)	84 (19.4)	146 (16.1)	98 (15.4)	48 (17.6)	
Other	249 (3.1)	22 (5.1)	42 (4.6)	19 (3.0)	23 (8.4)	
Class of stenosis						0.0
Type A	972 (12.0)	49 (11.3)	119 (13.1)	85 (13.4)	34 (12.5)	
Type B1	3146 (38.8)	157 (36.3)	351 (38.6)	245 (38.5)	106 (38.8)	
Type B2	2634 (32.5)	137 (31.7)	280 (30.8)	195 (30.7)	85 (31.1)	
Type C	1359 (16.8)	89 (20.6)	159 (17.5)	111 (17.5)	48 (17.6)	
Treated vessel						0.0
RCA	2734 (33.7)	120 (27.8)	258 (28.4)	168 (26.4)	90 (33.0)	
Left main	93 (1.2)	3 (0.7)	9 (1.0)	7 (1.1)	2 (0.7)	
LAD	3673 (45.3)	246 (56.9)	447 (49.2)	316 (49.7)	131 (48.0)	
LCX	1381 (17.0)	54 (12.5)	166 (18.3)	130 (20.4)	36 (13.2)	
Other	230 (2.8)	9 (2.1)	29 (3.2)	15 (2.4)	14 (5.1)	
Type of device(s) used*						0.0
BMS only	671 (8.3)	30 (6.9)	74 (8.1)	51 (8.0)	23 (8.4)	
BMS, predilation with balloon	1388 (17.1)	95 (22.0)	131 (14.4)	93 (14.6)	38 (13.9)	
DES only	1302 (16.1)	52 (12.0)	151 (16.6)	114 (17.9)	37 (13.6)	
DES, predilation with balloon	4124 (50.8)	216 (50.0)	483 (53.1)	331 (52.0)	152 (55.7)	
Balloon only, drug‐coated	138 (1.7)	8 (1.9)	17 (1.9)	11 (1.7)	6 (2.2)	
Balloon only, not drug‐coated	488 (6.0)	31 (7.2)	53 (5.8)	36 (5.7)	17 (6.2)	
Length of stent(s) if used (SD)	19.3 (7.5)	19.6 (7.8)	19.2 (7.1)	19.0 (6.8)	19.9 (7.9)	0.0
Stent diameter >3 mm if stent used	1994 (26.6)	105 (26.7)	244 (29.1)	166 (28.2)	78 (31.2)	0.0

BMS indicates bare metal stents; CAD, coronary artery disease; DES, drug eluting stent; DTD, drug treated device; LAD, left anterior descending coronary artery; LCX, left circumflex artery; MI, myocardial infarction; NSTEMI, non–ST‐segment–elevation myocardial infarction; PCI, percutaneous coronary intervention; RCA, right coronary artery; and STEMI, ST‐segment–elevation myocardial infarction.

* Bare metal stent categories include 15 segments in total with “Other” type of stent.

## RESULTS

Our final sample consisted of 6065 patients with 9452 segments. Median time between first pregnancy and the index PCI procedure was 30.9 years (interquartile range, 25.0–35.4). Patient‐ and procedural‐characteristics per segment, by obstetric history, are listed in Table 1. For patient‐ and procedural‐characteristics per‐patient see Table S1. There were no major differences in procedural characteristics between the preeclampsia exposure groups. Women with a history of preeclampsia were younger than women with normotensive pregnancies. Compared with women with normotensive pregnancies, women with a history of preeclampsia or hypertension in pregnancy more often had diabetes and hypertension, and they were less likely to smoke at time of index‐PCI.

**Figure 2:**
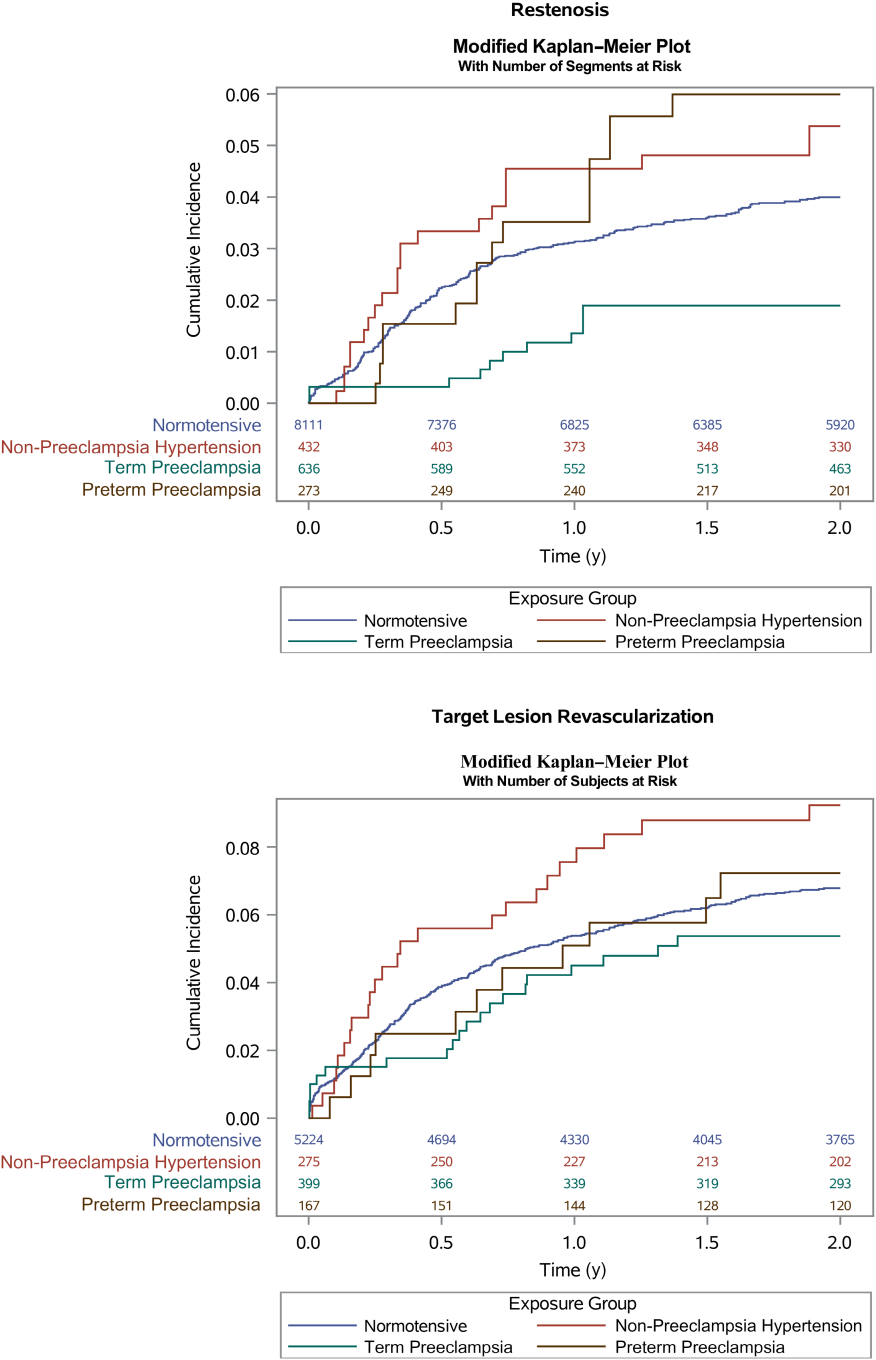
Modified Kaplan–Meier curves of restenosis and target lesion revascularization within 2 years by history of preeclampsia.

During 16 084 segment‐years of follow‐up, 345 restenosis events occurred, translating to 2.1% restenosis rate per year of follow‐up and 3.7% absolute risk within 2 years. In Figure 2, we show Kaplan–Meier curves of time to restenosis by exposure to preeclampsia. In Table 2 we show HRs for restenosis by preeclampsia history. In the model with all predictors, a history of preeclampsia was not significantly associated with a higher risk of restenosis compared with women with normotensive pregnancies (0.71, [95% CI, 0.41–1.23]). A history of term preeclampsia was significantly associated with a lower risk (0.45; [95% CI, 0.21–0.94]). In general, including established predictors of restenosis in modeling resulted only in minor changes of preeclampsia history HRs. Similar results as those presented here were observed in the complete case analyses (Table S2).

**Table 2:** Risk for Restenosis After PCI by Preeclampsia History in the Per‐Segment Analysis

				Subgroups of preeclampsia	
	Normotensive	Nonpreeclampsia hypertension in pregnancy	Preeclampsia	Term preeclampsia	Preterm preeclampsia
Events/Segment‐years	297/13 758	22/753	26/1573	11/1104	15/469
		HR (95% CI)	HR (95% CI)	HR (95% CI)	HR (95% CI)
Model I	1 (Reference)	1.34 (0.75–2.40)	0.73 (0.42–1.25)	0.45 (0.22–0.95)*	1.33 (0.62–2.86)
Model II	1 (Reference)	1.20 (0.67–2.16)	0.77 (0.45–1.32)	0.47 (0.23–0.98)*	1.47 (0.68–3.15)
Model III	1 (Reference)	1.15 (0.64–2.07)	0.71 (0.41–1.23)	0.45 (0.21–0.94)*	1.28 (0.59–2.80)

* Hazard ratios denote *P* value <0.05.

BMS indicates bare metal stents; CAD, coronary artery disease; DES, drug‐eluting stent; HR, hazard ratio; LAD, left anterior descending coronary artery; LCX, left circumflex artery; MI, myocardial infarction; NSTEMI, non–ST‐segment–elevation myocardial infarction; PCI, percutaneous coronary intervention; RCA, right coronary artery; and STEMI, ST‐segment–elevation myocardial infarction. Model I: age at index percutaneous coronary intervention. Model II: additionally accounted for indication of percutaneous coronary intervention (STEMI, NSTEMI, unstable coronary artery disease, stable coronary artery disease, other); year of procedure (2006–2009, 2010–2013, 2014–2017); treated vessel (right coronary artery, left main, left anterior descending coronary artery, left circumflex artery, other); class of stenosis (A, B1, B2, or C); type of device(s) (bare metal stents only, [bare metal stents, predilation with balloon], drug‐eluting stent only, [drug‐eluting stent, predilation with balloon], [balloon only, drug‐coated], or [balloon only, not drug‐coated]); length of stent; stent diameter >3 mm. Model III: additionally accounted for diabetes; hypertension; dyslipidemia; smoking; previous myocardial infarction.

In the TLR (per‐patient) analysis 383 events occurred during 10 174 person‐years, translating to 3.8% TLR rate per year of follow‐up or 6.3% absolute risk within 2 years. In Table 3, we show that there was a nominally modest reduced HR for TLR by exposure to preeclampsia (0.74, [95% CI, 0.51–1.07]) in the model accounting for all predictors with lower estimates for term preeclampsia compared with preterm preeclampsia. Similar results as those presented here were observed in the complete case analysis (Table S3).

**Table 3:** Risk for Target Lesion Revascularization by Preeclampsia History in the Per‐Patient Analysis

	Normotensive	Nonpreeclampsia hypertension in pregnancy	Preeclampsia	Subgroups of preeclampsia	
				Term preeclampsia	Preterm preeclampsia
Events/Person‐years	328/8745	24/464	31/965	20/683	11/282
		HR (95% CI)	HR (95% CI)	HR (95% CI)	HR (95% CI)
Model I	1 (Reference)	1.37 (0.90–2.07)	0.82 (0.57–1.19)	0.77 (0.49–1.20)	0.95 (0.52–1.74)
Model II	1 (Reference)	1.23 (0.81–1.87)	0.80 (0.55–1.16)	0.74 (0.47–1.16)	0.96 (0.52–1.76)
Model III	1 (Reference)	1.14 (0.75–1.74)	0.74 (0.51–1.07)	0.69 (0.44–1.09)	0.85 (0.46–1.57)

BMS indicates bare metal stents; CAD, coronary artery disease; DES, drug‐ eluting stent; HR, hazard ratio; LAD, left anterior descending coronary artery; LCX, left circumflex artery; MI, myocardial infarction; NSTEMI, non–ST‐segment–elevation myocardial infarction; PCI, percutaneous coronary intervention; RCA, right coronary artery; and STEMI, ST‐segment–elevation myocardial infarction.

Model I: age at index percutaneous coronary intervention. Model II: additionally accounted for indication of percutaneous coronary intervention (STEMI, NSTEMI, unstable coronary artery disease, stable coronary artery disease, other); year of procedure (2006–2009, 2010–2013, 2014–2017); right coronary artery‐treated; left main‐treated; left anterior descending coronary artery–treated; left circumflex artery–treated; other vessel–treated; number of treated vessels (1, 2, or ≥3). Model III: additionally accounted for diabetes; hypertension; dyslipidemia; smoking; previous myocardial infarction.

In the all‐cause mortality analysis 166 events occurred during 10 684 person‐years, translating to 1.55% rate of death per year. Neither history of preeclampsia (HR, 1.06 [95% CI, 0.62–1.80]) nor non‐preeclamptic hypertension in pregnancy (HR, 1.21 [95% CI, 0.62–2.38]) were significantly associated with death within 2 years from the index PCI.

As an analytic validation, the univariable associations between the clinical predictors of restenosis and actual restenosis risk is presented in Table S4 and Table S5. In general, these associations presented as expected, eg, diabetes and undergoing the index procedure early in the study period were associated with an increased risk of clinical restenosis.

## DISCUSSION

In contrast to our hypothesis, we did not observe a higher risk of neither restenosis nor need for TLR in women with a history of preeclampsia. Instead, we found some evidence indicating a lower risk of restenosis in women with a history of preeclampsia, with the rate of restenosis being substantially lower in coronary artery segments of patients with a history of term preeclampsia. However, this per‐segment finding did not translate into a dramatically lower risk of TLR in patients with a history of term (or any) preeclampsia, despite similar numbers of outcome events.

According to the most widely accepted hypothesis, the cause of preeclampsia somewhat differs according to its debut during pregnancy.^16^, ^17^ Preterm preeclampsia is thought to be related to suboptimal cardiovascular adaptation to pregnancy and placental implantation whereas preeclampsia at term is considered to a larger extent to be caused by metabolic stress inflicted on the placenta in late pregnancy. Our hypothesis that preeclampsia history would be associated with increased risk of restenosis is derived from animal models in which excessive exposure to the Sflt‐1 receptor, a likely downstream mediator of preeclamptic symptoms as an antagonist of vascular endothelial growth factor, caused long‐term disruption of healing following vascular injury.^8^ Mean Sflt‐1 level during pregnancy is higher in women with preeclampsia and Sflt‐1 level seems to correlate with severity of disease,^8^ but women can also have high Sflt‐1 without developing preeclampsia.^18^ In addition, recent studies have shown that women with preeclampsia or hypertensive disorders of pregnancy in multi‐fetal pregnancies have lower risk of incident cardiovascular disease compared with women with a history of preeclampsia in singleton pregnancies^19^, ^20^ despite having high levels of Sflt‐1. This could suggest that the vascular dysfunction observed following preeclampsia is not because of Sflt‐1 exposure but rather because of a shared antecedent of preeclampsia and disturbed vascular function in singleton pregnancies, whilst in twin pregnancies preeclampsia is caused by the greater pregnancy burden. Regardless, our results indicate that coronary artery segments of women with a history of term preeclampsia may respond differently to treatment with PCI compared with those in women without such history.

As our study is based on clinical registry data, the pathophysiological basis of why women with a history of preeclampsia would have lower risk of restenosis remains elusive. However, we hypothesize that this could be explained by a difference in endothelial progenitor cells by preeclampsia history. Based on the important role of endothelial progenitor cells in vascular healing,^21^ stents capturing endothelial progenitor cells were developed to lower risk of in‐stent restenosis and thrombosis. The Japan‐USA harmonized assessment by randomized multicentre study of Orbusneich's combo stent (HARMONEE) trial reported higher strut coverage at 1 year in patients with a DES capturing endothelial progenitor cells compared with a regular DES.^22^ However, the Tri‐stent adjudication study–high risk of restenosis (TRIAS‐HR) trial was stopped early because of a high risk of target lesion failure in patients with stents attracting endothelial progenitor cells compared with DES^23^ and the Combo stent versus Orsiro stent (SORT OUT X) trial reported higher target lesion failure for a DES capturing endothelial progenitor cells compared with a regular DES.^24^ While the level of endothelial progenitor cells increases with gestational age in normal pregnancies,^25^ some studies suggest that endothelial progenitor cell levels are lower in women who develop preeclampsia.^26^, ^27^ If women with a history of preeclampsia also have a different level or function of endothelial progenitor cells following PCI compared with patients with only normotensive pregnancies, it might affect their risk of restenosis.

The clinical results in interventional cardiology are affected by the tradeoff between stent thrombosis, restenosis, and bleeding.^28^ Thus, the identification of subgroups of patients with different optima in this tradeoff constitutes an important step to improve patient outcome by implementing specific treatment strategies. Speculatively, a subgroup with reduced preponderance of restenosis might benefit from receiving a stent with shorter time to strut coverage, shorter time on anti‐thrombotic therapy, and, as a result, shorter time with increased bleeding risk. Diabetes is a component of a patient's medical history already used to inform revascularization decisions, to a larger extent favoring CABG rather than PCI in some patients.^29^, ^30^

### Strengths and Limitations

The strengths of this study include the comprehensive study sample with data collected from nationwide clinical registries covering almost all deliveries and all PCIs performed in Sweden. This allowed us to consider a range of relevant procedural‐ and patient‐related predictors of restenosis in our analysis. Furthermore, we used multiple imputation to account for a small proportion of missing covariable data to avoid exclusion of otherwise eligible patients from the study. However, there are also limitations to our study that should be acknowledged. Firstly, the validity of our results is contingent on that there is no major residual difference in PCI‐related care (eg, drug treatment) that is relevant for clinical restenosis by history of preeclampsia. We consider such difference to be unlikely as we observed no major difference in the restenosis estimates by preeclampsia history when we accounted for several important procedural‐ and patient‐related predictors. Secondly, our results mainly pertain to the younger age span of women undergoing PCI as we study women aged ≤65 years. We consider this age restriction necessary for study validity as the delivery registry started in the early 1970s, and older women to a much lesser extent therefore have complete preeclampsia history data available. Still, young‐ and middle‐aged women (aged <65 years) have increased risk of major adverse cardiovascular events following PCI compared with older women, which seems to partly be explained by a higher incidence of repeat revascularization.^31^ Given that the association between pregnancy‐related complications and cardiovascular disease seems to be attenuated with age,^32^ we speculate that the associations investigated in this study should be attenuated, rather than augmented, in older women. Thirdly, in our definition of TLR we include all types of CABG regardless of targeted vessels. Lastly, it should be noted that the absolute number of restenosis events in women with a history of preeclampsia was small resulting in wide CIs.

In conclusion, a history of preeclampsia was not associated with increased risk of restenosis after PCI but instead some evidence pointed to reduced risk. However, data on complete pregnancy complication history are seldom available in studies of women undergoing PCI. To facilitate future studies and allow for replication, concomitant collection of data on pregnancy complication history and PCI outcomes in women is warranted.

## Sources of Funding

This work was funded by the Swedish Research Council (2019–02082); Jeansson Foundations, Gothenburg, Sweden; Åke Wiberg Foundation, Stockholm, Sweden; The Swedish Heart‐Lung Foundation (20180312); Crafoord Foundation, Lund Sweden; The Swedish Society of Medicine, and research support from the health care authority in Region Skåne, Sweden (regional funds and YF‐ALF).

## Disclosures

None.

## Supporting information

Data S1
Tables S1–S5
