# Supplementary material for: Coronary Artery Restenosis in Women by History of Preeclampsia
Source: J Am Heart Assoc. 2022 Sep 8;11(18):e026287. doi: 10.1161/JAHA.122.026287 (PMC9683658; doi:10.1161/JAHA.122.026287)
Supplement: Supplementary file 1 — Data S1 Tables S1–S5 [file JAH3-11-e026287-s001.pdf]

# SUPPLEMENTAL MATERIAL

**Data S1.**

## Supplemental Methods

### ICD codes for hypertensive disorders of pregnancy:

ICD 8 used during the years 1973-1986

ICD 9 used during the years 1987-1996

ICD 10 used from 1997 and forward.

We defined PE as 637.03, 637.04, 637.09, and 637.10 when using ICD 8; 642E, 642F, and 642G when using ICD 9; O14 and O15 when using ICD 10. Non-preeclamptic hypertension in pregnancy was defined as 637.01 when using ICD 8, 642D or 642X when using ICD 9, O13.9 when using ICD 10.

**Table S1. Descriptive characteristics per patient (n = 6,065) at index PCI procedure by preeclampsia history**

| Variables n, (%) unless stated | Normotensive (n=5,224) | Non-PE hypertension in pregnancy (n=275) | PE (n=566) | Subgroups of PE: Term PE (n=399) | Subgroups of PE: Preterm PE (n=167) | Missing (%) |
|---|---|---|---|---|---|---|
| **Patient characteristics** | | | | | | |
| Age (SD) | 55.3 (6.3) | 54.8 (6.5) | 53.0 (7.2) | 54.0 (6.8) | 50.5 (7.6) | 0.0 |
| Previous MI | 198 (3.8) | 16 (6.0) | 20 (3.6) | 9 (2.3) | 11 (6.7) | 1.2 |
| Diabetes | 743 (14.3) | 67 (24.7) | 144 (25.5) | 88 (22.2) | 56 (33.5) | 0.5 |
| Hypertension | 2,154 (41.9) | 175 (64.8) | 345 (61.4) | 242 (61.3) | 103 (61.7) | 1.5 |
| Dyslipidemia | 1,538 (30.0) | 99 (37.2) | 193 (34.6) | 130 (33.1) | 63 (38.2) | 1.7 |
| Smoking | | | | | | 3.6 |
| Never | 1,380 (27.4) | 99 (38.2) | 229 (41.8) | 156 (40.2) | 73 (45.6) | |
| Ex-smoker | 1,215 (24.1) | 71 (27.4) | 150 (27.4) | 114 (29.4) | 36 (22.5) | |
| Smoker | 2,446 (48.5) | 89 (34.4) | 169 (30.8) | 118 (30.4) | 51 (31.9) | |
| **Procedural characteristics** | | | | | | |
| Year of index PCI | | | | | | 0.0 |
| 2006 - 2009 | 1,319 (25.3) | 92 (33.5) | 150 (26.5) | 103 (25.8) | 47 (28.1) | |
| 2010 - 2013 | 1,762 (33.7) | 98 (35.6) | 190 (33.6) | 141 (35.3) | 49 (29.3) | |
| 2014 - 2017 | 2,143 (41.0) | 85 (30.9) | 226 (39.9) | 155 (38.9) | 71 (42.5) | |
| Indication for PCI | | | | | | 0.0 |
| STEMI | 1,935 (37.0) | 100 (36.4) | 182 (32.2) | 125 (31.3) | 57 (34.1) | |
| NSTEMI | 724 (13.9) | 31 (11.3) | 77 (13.6) | 59 (14.8) | 18 (10.8) | |
| Unstable CAD | 1,672 (32.0) | 84 (30.6) | 193 (34.1) | 144 (36.1) | 49 (29.3) | |
| Stable CAD | 732 (14.0) | 49 (17.8) | 91 (16.1) | 59 (14.8) | 32 (19.2) | |
| Other | 161 (3.1) | 11 (4.0) | 23 (4.1) | 12 (3.0) | 11 (6.6) | |
| RCA treated | 1,892 (36.2) | 78 (28.4) | 173 (30.6) | 117 (29.3) | 56 (33.5) | 0.0 |
| Left main treated | 85 (1.6) | 3 (1.1) | 8 (1.4) | 6 (1.5) | 2 (1.2) | 0.0 |
| LAD treated | 2,645 (50.6) | 171 (62.2) | 329 (58.1) | 230 (57.6) | 99 (59.3) | 0.0 |
| LCX treated | 1,119 (21.4) | 47 (17.1) | 128 (22.6) | 98 (24.6) | 30 (18.0) | 0.0 |
| Other vessel treated | 213 (4.1) | 9 (3.3) | 26 (4.6) | 14 (3.5) | 12 (7.2) | 0.0 |
| N treated vessels | | | | | | |
| 1 | 3,301 (63.2) | 166 (60.4) | 341 (60.3) | 242 (60.6) | 99 (59.3) | 0.0 |
| 2 | 1,267 (24.3) | 77 (28.0) | 148 (26.2) | 104 (26.1) | 44 (26.4) | |
| ≥3 | 656 (12.6) | 32 (11.6) | 77 (13.6) | 53 (13.3) | 24 (14.4) | |

BMS: bare metal stents; CAD: coronary artery disease; DES: drug eluting stent; LAD: left anterior descending coronary artery; LCX: left circumflex artery; MI: myocardial infarction; NSTEMI: non ST-elevation myocardial infarction; PE: preeclampsia; PCI: percutaneous coronary intervention; RCA: right coronary artery; STEMI: ST-elevation myocardial infarction

**Table S2. Complete case analysis of risk for restenosis per-segment after PCI by preeclampsia history**

| | Normotensive | Non-PE hypertension in pregnancy | PE | Subgroups of PE | |
|---|---|---|---|---|---|
| | | | | **Term PE** | **Preterm PE** |
| Events/ Person years | 279/13,094 | 20/705 | 20/1,506 | 10/1,062 | 10/444 |
| | | **HR (95% CI)** | **HR (95% CI)** | **HR (95% CI)** | **HR (95% CI)** |
| Model I | 1 (Reference) | 1.32 (0.72, 2.42) | 0.59 (0.34, 1.04) | **0.43 (0.20, 0.95)** | 0.95 (0.43, 2.11) |
| Model II | 1 (Reference) | 1.18 (0.64, 2.16) | 0.62 (0.36, 1.10) | **0.45 (0.21, 0.98)** | 1.04 (0.47, 2.29) |
| Model III | 1 (Reference) | 1.14 (0.62, 2.10) | 0.59 (0.33, 1.03) | **0.43 (0.20, 0.96)** | 0.92 (0.42, 2.00) |

HRs in bold are p-values <0.05

BMS: bare metal stents; CAD: coronary artery disease; DES; drug eluting stent; HR: Hazard ratio; LAD: left anterior descending coronary artery; LCX: left circumflex artery; MI: myocardial infarction; NSTEMI: non ST-elevation myocardial infarction; PE: preeclampsia; PCI: percutaneous coronary intervention; RCA: right coronary artery; STEMI: ST-elevation myocardial infarction

Model I: age at index PCI

Model II: additionally accounted for indication of PCI (STEMI, NSTEMI, unstable CAD, stable CAD, other); year of procedure (2006 – 2009, 2010 – 2013, 2014 – 2017); treated vessel (RCA, left main, LAD, LCX, other); class of stenosis (A, B1, B2, or C); type of device(s) (BMS only, [BMS, predilation with balloon], DES only, [DES, predilation with balloon], [Balloon only, drug coated], or [Balloon only, not drug coated]); length of stent; stent diameter >3mm

Model III: additionally accounted for diabetes; hypertension; dyslipidemia; smoking; previous MI

**Table S3. Complete case analysis of risk for target lesion revascularization after PCI per-patient by preeclampsia history**

| | | | | Subgroups of PE | |
|---|---|---|---|---|---|
| | **Normotensive** | **Non-PE hypertension in pregnancy** | **PE** | **Term PE** | **Preterm PE** |
| Events/ Person years | 305/8,335 | 22/430 | 28/928 | 19/661 | 9/267 |
| | | **HR (95% CI)** | **HR (95% CI)** | **HR (95% CI)** | **HR (95% CI)** |
| Model I | 1 (Reference) | 1.39 (0.90, 2.14) | 0.80 (0.54, 1.17) | 0.78 (0.49, 1.23) | 0.84 (0.43, 1.64) |
| Model II | 1 (Reference) | 1.26 (0.81, 1.94) | 0.78 (0.53, 1.15) | 0.75 (0.47, 1.19) | 0.85 (0.43, 1.66) |
| Model III | 1 (Reference) | 1.18 (0.76, 1.83) | 0.72 (0.49, 1.07) | 0.70 (0.44, 1.13) | 0.77 (0.39, 1.51) |

BMS: bare metal stents; CAD: coronary artery disease; DES; drug eluting stent; HR: Hazard ratio; LAD: left anterior descending coronary artery; LCX: left circumflex artery; MI: myocardial infarction; NSTEMI: non ST-elevation myocardial infarction; PE: preeclampsia; PCI: percutaneous coronary intervention; RCA: right coronary artery; STEMI: ST-elevation myocardial infarction

Model I: age at index PCI

Model II: additionally accounted for indication of PCI (STEMI, NSTEMI, unstable CAD, stable CAD, other); year of procedure (2006 – 2009, 2010 – 2013, 2014 – 2017); RCA treated; Left main treated; LAD treated; LCX treated; Other vessel treated; number of treated vessels (1, 2 or ≥3)

Model III: additionally accounted for diabetes; hypertension; dyslipidemia; smoking; previous MI

**Table S4. Associations between predictors of restenosis and risk of clinical restenosis per-segment**

| Predictor | Unit, comparison, or categories | Hazard ratio (95% CIs) | *p* overall association |
|---|---|---|---|
| Age | | | 0.057 |
| | Years | 0.98 (0.96, 1.00) | |
| Previous MI | | | 0.26 |
| | Yes vs. no | 1.39 (0.79, 2.43) | |
| Diabetes | | | 0.011 |
| | Yes vs. no | 1.55 (1.10, 2.17) | |
| Hypertension | | | 0.70 |
| | Yes vs. no | 0.94 (0.70, 1.27) | |
| Dyslipidemia | | | 0.028 |
| | Yes vs. no | 1.40 (1.04, 1.89) | |
| Smoking | | | |
| | Never | 1 (Reference) | 0.52 |
| | Ex-smoker | 0.99 (0.66, 1.47) | |
| | Smoker | 0.84 (0.60, 1.18) | |
| Year of index PCI | | | <0.0001 |
| | 2006 – 2009 | 1 (Reference) | |
| | 2010 – 2013 | 0.58 (0.42, 0.81) | |
| | 2014 – 2017 | 0.28 (0.19, 0.42) | |
| Indication for PCI | | | 0.085 |
| | STEMI | 1 (Reference) | |
| | NSTEMI | 0.50 (0.24, 1.04) | |
| | Unstable CAD | 1.33 (0.94, 1.88) | |
| | Stable CAD | 1.12 (0.74, 1.68) | |
| | Other | 0.96 (0.42, 2.18) | |
| Class of stenosis | | | 0.63 |
| | Type A | 1 (Reference) | |
| | Type B | 1.05 (0.72, 1.53) | |
| | Type B2 | 1.12 (0.78, 1.80) | |
| | Type C | 1.28 (0.81, 2.02) | |
| Treated vessel | | | 0.134 |
| | LAD | 1 (Reference) | |
| | Left main stem | 1.04 (0.31, 3.43) | |
| | LCX | 0.73 (0.51, 1.04) | |
| | RCA | 0.66 (0.47, 0.93) | |
| | Other | 0.72 (0.35, 1.47) | |
| Type of device(s) used* | | | <0.0001 |
| | BMS only | 1 (Reference) | |
| | BMS, predilation with balloon | 1.29 (0.90, 1.86) | |
| | DES only | 0.42 (0.25, 0.69) | |
| | DES, predilation with balloon | 0.38 (0.25, 0.58) | |
| | Balloon only, drug coated | 1.00 (0.43, 2.31) | |
| | Balloon only, not drug coated | 1.12 (0.71, 1.76) | |
| Length of stent(s) | | | 0.005 |
| | Mm | 0.98 (0.97, 0.99) | |
| Stent diameter >3 mm | | | 0.001 |
| | Yes vs. no | 0.73 (0.53, 0.99) | |

All predictors are analyzed in a univariable complete case analysis using proportional hazards regression with a jackknife estimator of variance and 24 months of follow-up.
BMS: bare metal stents; CAD: coronary artery disease; CI: Confidence interval; DES: drug eluting stent; HR: hazard ratio; LAD: left anterior descending coronary artery; LCX: left circumflex artery; MI: myocardial infarction; NSTEMI: non ST-elevation myocardial infarction; PE: preeclampsia; PCI: percutaneous coronary intervention; RCA: right coronary artery; STEMI: ST-elevation myocardial infarction
∗ BMS categories includes 15 segments in total with “Other” type of stent.

**Table S5. Associations between predictors of restenosis and risk of target lesion revascularization per-patient**

| Predictor | Unit, comparison, or categories | Hazard ratio (95% CIs) | *p* overall association with restenosis |
|---|---|---|---|
| Age | | | 0.033 |
| | Years | 0.98 (0.97, 1.00) | |
| Previous MI | | | 0.28 |
| | Yes vs. no | 1.30 (0.81, 2.09) | |
| Diabetes | | | 0.053 |
| | Yes vs. no | 1.30 (1.00, 1.69) | |
| Hypertension | | | 0.22 |
| | Yes vs. no | 1.14 (0.93, 1.40) | |
| Dyslipidemia | | | 0.14 |
| | Yes vs. no | 1.18 (0.95, 1.47) | |
| Smoking | | | 0.15 |
| | Never | 1 (Reference) | |
| | Ex-smoker | 1.00 (0.76, 1.31) | |
| | Smoker | 0.81 (0.64, 1.04) | |
| Year of index PCI | | | <0.0001 |
| | 2006 - 2009 | 1 (Reference) | |
| | 2010 - 2013 | 0.67 (0.52, 0.85) | |
| | 2014 - 2017 | 0.44 (0.34, 0.58) | |
| Indication for PCI | | | 0.0064 |
| | STEMI | 1 (Reference) | |
| | NSTEMI | 0.51 (0.33, 0.80) | |
| | Unstable CAD | 1.14 (0.89, 1.45) | |
| | Stable CAD | 1.15 (0.84, 1.56) | |
| | Other | 1.28 (0.71, 2.32) | |
| Treated vessel | | | <0.0001 |
| LAD | Yes vs. no | 1.52 (1.23, 1.89) | |
| Left main | Yes vs. no | 2.53 (1.42, 4.50) | |
| LCX | Yes vs. no | 1.00 (0.77, 1.29) | |
| RCA | Yes vs. no | 0.74 (0.59, 0.93) | |
| Other | Yes vs. no | 1.16 (0.71, 1.89) | |
| N treated vessels | | | <0.0001 |
| | 1 | 1 (Reference) | |
| | 2 | 1.51 (1.19, 1.91) | |
| | ≥3 | 1.83 (1.31, 2.56) | |

All predictors are analyzed in a univariable complete case analysis using proportional hazards regression and 24 months of follow-up.
BMS: bare metal stents; CAD: coronary artery disease; DES: drug eluting stent; LAD: left anterior descending coronary artery; LCX: left circumflex artery; MI: myocardial infarction; NSTEMI: non ST-elevation myocardial infarction; PE: preeclampsia; PCI: percutaneous coronary intervention; RCA: right coronary artery; STEMI: ST-elevation myocardial infarction
